# Practice postcode versus patient population: a comparison of data sources in England and Scotland

**DOI:** 10.1186/1476-072X-7-37

**Published:** 2008-07-16

**Authors:** Gary McLean, Bruce Guthrie, Graham Watt, Mark Gabbay, Catherine A O'Donnell

**Affiliations:** 1 General Practice and Primary Care, Division of Community-based Sciences, University of Glasgow, Glasgow, 1 Horselethill Road Glasgow, G12 9LX, UK; 2 Tayside Centre for General Practice, University of Dundee, The Mackenzie Building, Kirsty Semple Way, DD2 4BF, UK; 3 Division of Primary Care, Brownlow Hill, University of Liverpool, L69 3GB, UK

## Abstract

**Background:**

Health professionals, policy-makers and researchers need to be able to explore potential associations between prevalence rates and quality of care with a range of possible determinants including socio-economic deprivation and morbidity levels to determine the impact of commissioning and service delivery. In the UK, data in England are only available nationally at practice postcode level. In Scotland, such data are available based on an aggregate of the practices population's postcodes. The use of data assigned to the practice postcode may underestimate the association between ill health and income deprivation. Here, we report on the impact of using data assigned to the practice population by comparing analyses using English and Scottish data.

**Results:**

Income deprivation based on data assigned to the practice postcode under-estimated deprivation compared to using income deprivation data assigned to the practice population for the five least deprived deciles, and over-estimated deprivation for the five most deprived deciles. The biggest differences were found for the most deprived decile. A similar trend was found for limiting long-term illness (LLTI). Differences between the QOF prevalence rates of the least and most deprived deciles using practice postcode data were similar (0.2% points or less) in England and Scotland for 8 out of 10 clinical domains. Using practice population assigned deprivation, differences in the prevalence rate between the least and most deprived deciles increase for all clinical domains. A similar trend was again found for LLTI. Using practice population assigned deprivation, differences for population achievement increase for all CHD quality indicators with the exception of beta-blockers (CHD10). With practice postcode assigned deprivation, significant differences between the least and most deprived deciles were found for 2 out 8 indicators, compared to 5 using practice population assigned deprivation. For LLTI differences between the lowest and most deprived deciles increased for all indicators when ill health assigned to the practice population was used.

**Conclusion:**

We have found, through comparing deprivation and ill health data assigned to either the practice postcode or the practice population postcode in Scotland, that analyses based on practice postcode assigned data under-estimated the relationship between deprivation and ill health for both prevalence and quality care. Given the importance of understanding the effect of deprivation and ill health on a range of determinants related to health care, policy makers should ensure that practice population data are available and used at national level in England and elsewhere where possible.

## Background

Health professionals, policy-makers and researchers need to be able to explore potential associations between prevalence rates and quality of care with a range of possible determinants including socio-economic deprivation and morbidity levels to determine the impact of commissioning and service delivery. When undertaking such research it is important to understand the ways in which data are collected and how this may impact on the possible interpretations of the analysis [1]. Assessment of whether the results produced are a reasonable representation of what is actually happening, requires a comprehensive knowledge of the strengths and weakness of the data, [2] particularly when results are being compared across countries and health care systems. The Quality Outcomes Framework (QOF) forms a major part of the General Medical Services (GMS) contract implemented across the United Kingdom from April 2004 under which up to a quarter of General Practitioners' (GP) income depends on practice performance measured against 146 clinical and organisational indicators. This provides a new opportunity to compare prevalence and clinical quality using measures with consistent definitions and data collection methods across the UK [3,4]. Research undertaken on the QOF has examined differences in quality of care in England and in Scotland by deprivation [5-7] practice characteristics [8-10] remoteness [7,11] and ill health [12]. Further work has used a combined English and Scottish dataset to explore the association between quality of care for cardiovascular disease (CVD) by general practice caseload, practice size and deprivation [13] and examined the quality of care across the four UK countries for CVD and diabetes [4]. However, there has been no research on the QOF that has compared differences between England and Scotland in terms of how prevalence and quality of care vary by deprivation and ill health.

One of the most important considerations in undertaking comparative analysis is to ensure that data are collected and measured in a similar way. Whilst data on the QOF indicators are collected in a standardised manner across the UK, data on population socio-economic variables or the structural characteristics of practices are often not comparable, for example because of varying data definitions or because data may simply not be collected at national level in every country. It is possible to examine how determinants such as deprivation and ill health impact on disease prevalence and the quality of health care, as measured in the QOF, by using variables that are constructed in a comparable manner for England and Scotland. However, this does not tell us how differences in data definitions and collection may affect our interpretation of the impact of such variables on prevalence and quality of care. Understanding such differences in the data is important, as inappropriate or poorer data sources can add to measurement concerns which may lead to the over or underestimation of the associations being explored [1].

Compared to the rest of the UK, Scotland has historically had more complete and comprehensive approach to the collection of routine data largely through the Information and Statistics Division (ISD) of NHS Scotland [14]. One particular area where Scotland has an advantage over England is in the availability of a number of indicators based on the registered practice population, referred to by others as the 'gold standard' method [15]. Unlike Scotland, data about practice populations in England are often only available nationally at the level of the postcode for where the practice itself is located. While this method is seen as being a valid proxy for a population weighted measure in the absence of patient-level data, it has been found, at the level of a Primary Care Trust (PCT) in England, to underestimate the association between ill-health and deprivation when compared to results based on registered practice population datasets [16]. Using data at practice postcode level allows for a direct comparison between England and Scotland on a range of variables. However, within Scotland we can further compare findings based on practice postcode level data with those obtained using practice population data, and use those findings to help interpret associations between prevalence and quality, and practice postcode level explanatory variables in England. For example, does the use of data at the level of the postcode in which the practice is located overestimate or underestimate the association between QOF points' achievement and deprivation.

This paper aims to examine the impact of the level at which socio-economic and health data are linked in terms of estimating the association between these variables and disease prevalence and quality of care, by comparing practice and population-level data from Scotland. It also aims to explore how England and Scotland compare by deprivation and ill health in QOF prevalence rates and achievement for Coronary Heart Disease (CHD) quality indicators and to interpret English results in light of any differences found between practice and population postcode level data in Scotland.

## Results

Table 1 shows mean deprivation and LLTI scores for deciles based on practice postcode assigned deprivation and LLTI for England and Scotland, as well as on practice population assigned deprivation and LLTI for Scotland only. For deprivation, similar scores were observed for England and Scotland using practice postcode data, with Scotland marginally higher for all but the least deprived decile. Deprivation results based on practice population data increased the mean scores for the lowest five deprived deciles but reduced them for the five most deprived deciles. The biggest difference was found for the most deprived decile with practice postcode assigned data overestimating income deprivation by 11.4 percentage points compared to practice population assigned data (practice postcode-assigned mean of 42.5% points vs. practice population-assigned mean of 31.1% points).

**Table 1:** Difference in mean IMD Income levels and long term limiting illness (LLTI)

**Decile**		**England** **Deciles based on** **practice postcode** ** assigned values**	**Scotland** **Deciles based on** **practice postcode** ** assigned values**	**Scotland** **Deciles based on** **practice population** ** assigned values**
		**Mean income score**	**Mean income score**	**Mean income score**
Least deprived decile	1	2.9	2.8	4.9
	2	5.0	5.6	7.7
	3	6.8	7.8	9.7
	4	8.7	10.0	11.2
	5	10.8	12.1	13.0
	6	13.2	15.1	15.0
	7	16.3	18.7	17.1
	8	20.7	22.7	19.4
	9	27.4	27.8	22.0
Most deprived decile	10	40.7	42.5	31.1

Ratio most:least deprived		14.0	14.0	6.3

		**Mean LLTI score**	**Mean LLTI score**	**Mean LLTI score**

Lowest LLTI decile	1	47.1	43.6	64.5
	2	65.3	59.2	75.3
	3	75.4	68.9	82.0
	4	83.5	78.7	88.4
	5	93.1	88.4	94.1
	6	98.2	98.5	100.1
	7	107.3	111.3	106.7
	8	121.4	125.8	112.8
	9	140.3	144.7	123.0
Highest LLTI decile	10	177.2	184.5	143.3

Ratio highest:lowest LLTI		3.8	4.2	2.2

Mean income score: % of patients receiving state benefits on the basis of low income. The higher the reported score, the more income deprived the practice population is.

Mean LLTI score: age-sex standardised ratio for limiting long-term illness. The higher the ratio, the greater the level of ill-health in the practice population.

A similar trend was found for LLTI. Using practice postcode data, England had higher mean LLTI scores for the lowest five deciles compared with Scotland, but this then reversed for the highest five deciles. Again, comparison of practice postcode level with practice population level found that scores assigned at the practice postcode level underestimated LLTI for the lowest six deciles but overestimated it for the highest four deciles. The biggest difference of 41.2 was found for the decile with the highest rates of LLTI (practice postcode-assigned mean of 184.5 vs. practice population-assigned mean of 143.3).

The impact of using practice or population assigned data was clearly illustrated by examining the ratio of deciles 10 to 1 (Table 1). For deprivation, the ratio between the most and last deprived deciles for England and Scotland was the same using practice postcode assigned data (14.0) but fell to 6.3 when using practice population assigned data for Scotland. For LLTI, the ratio between the highest and lowest LLTI deciles was similar for England and Scotland (3.8 and 4.2) using practice postcode assigned data but fell to 2.2 when using practice population assigned data for Scotland.

Table 2 shows the relationship between QOF prevalence rates and deprivation using practice postcode assigned data for England and Scotland and also practice population assigned data for Scotland. Differences between the least and most deprived deciles under practice postcode were similar (0.2% points or less) in England and Scotland for the majority of clinical domains. The exceptions were diabetes where the most deprived decile was 1.1% points higher in England, compared to 0.6% points in Scotland; hypertension (-0.2% points lower in England compared to 0.4% points higher in Scotland); and Chronic Obstructive Pulmonary Disease (COPD) (0.1% points higher in England compared to 1.1% points higher in Scotland). With practice population assigned data, differences between the least and most deprived deciles increased for all clinical domains. The largest variation between practice postcode and practice population data was found for COPD where predicted prevalence was 2.2% points higher in the most deprived decile compared to the least deprived decile using population postcode assigned data, but only 1.1% points higher using practice postcode assigned data. Differences were also found for CHD where predicted prevalence in the most deprived decile was 1.3% points higher using practice population assigned data compared to 0.8% points higher using practice postcode assigned data, and for asthma and hypothyroidism were the differences were 0.5 to 0.1 and -0.7 to -0.3 percentage points respectively.

**Table 2:** Differences between least and most deprived income deciles for QOF prevalence rates for practice

	**England: Prevalence rates based on ** **practice postcode**			**Scotland: Prevalence rates based on ** **practice postcode**			**Scotland: Prevalence rates based on ** **practice population**		
	**Least** **deprived** ** decile**	**Most** **deprived** ** decile**	**Difference**	**Least** **deprived** ** decile**	**Most** **deprived** ** decile**	**Difference**	**Least** **deprived** ** decile**	**Most** **deprived** ** decile**	**Difference**
CHD	3.1	3.7	0.6 [<0.001]	4.0	4.8	0.8 [<0.001]	3.7	5.0	1.3 [<0.001]
Diabetes	2.9	4.0	1.1 [<0.001]	3.0	3.6	0.6 [<0.001]	2.7	3.6	0.9 [<0.001]
Stroke	1.5	1.5	0.0 [0.79]	1.8	2.0	0.2 [0.07]	1.7	2.1	0.4 [<0.001]
Hypertension	11.5	11.3	-0.2 [0.39]	11.4	11.8	0.4 [0.50]	11.4	11.9	0.5 [0.24]
COPD	1.3	1.4	0.1 [0.04]	1.4	2.5	1.1 [<0.001]	0.9	3.1	2.2 [<0.001]
Asthma	5.8	5.7	-0.1 [0.73]	5.3	5.4	0.1 [0.19]	5.0	5.5	0.5 [0.01]
Cancer	0.7	0.6	-0.1 [<0.001]	0.8	0.7	-0.1 [0.03]	0.8	0.6	-0.2 [<0.001]
MH	0.6	0.7	0.1 [<0.001]	0.6	0.7	0.1 [0.10]	0.5	0.7	0.2 [<0.001]
Thyroid	2.4	2.2	-0.2 [<0.001]	2.9	2.6	-0.3 [<0.001]	3.1	2.4	-0.7 [<0.001]
Epilepsy	0.6	0.6	0.0 [0.96]	0.6	0.8	0.2 [<0.001]	0.5	0.9	0.4 [<0.001]

and population assigned data

Table 3 shows the relationship between QOF prevalence rates and LLTI using practice postcode assigned data for England and Scotland and practice population assigned data for Scotland. Differences in prevalence rates between the lowest and highest LLTI deciles in for practice postcode level showed a similar trend to that found for deprivation. For practice population assigned data, the difference between the lowest and highest LLTI deciles increased for all clinical domains. The biggest difference was again found for COPD where the difference was 1.1% points using practice postcode assigned data compared to 2.2% points using practice population assigned data. This was followed by diabetes (0.3 to 1.1% points) and CHD (0.9 to 1.6% points).

**Table 3:** Differences between lowest and highest deprived LLTI deciles for QOF prevalence rates for

	**England: Prevalence rates based on ** **practice postcode**			**Scotland: Prevalence rates based on ** **practice postcode**			**Scotland: Prevalence rates based on ** **practice population**		
	**Lowest** **LLTI** ** decile**	**Highest** **LLTI** ** decile**	**Difference**	**Lowest** **LLTI** ** decile**	**Highest** **LLTI** ** decile**	**Difference**	**Lowest** **LLTI** ** decile**	**Highest** **LLTI** ** decile**	**Difference**
CHD	3.2	3.8	0.6 [<0.001]	4.0	4.9	0.9 [<0.001]	3.5	5.1	1.6 [<0.001]
Diabetes	2.8	4.0	1.2 [<0.001]	3.1	3.4	0.3 [<0.001]	2.6	3.7	1.1 [<0.001]
Stroke	1.5	1.6	0.0 [0.84]	1.7	2.0	0.3 [0.01]	1.7	2.0	0.3 [0.01]
Hypertension	11.4	11.2	-0.2 [0.36]	11.1	11.9	0.8 [0.09]	10.9	12.1	1.2 [0.04]
COPD	1.2	1.4	0.1 [0.04]	1.3	2.4	1.1 [<0.001]	0.9	3.1	2.2 [<0.001]
Asthma	5.7	5.7	-0.1 [0.91]	5.2	5.4	0.2 [0.12]	5.1	5.5	0.4 [0.03]
Cancer	0.7	0.6	-0.1 [<0.001]	0.7	0.6	-0.1 [0.35]	0.7	0.6	-0.1 [0.04]
MH	0.7	0.7	0.1 [<0.001]	0.5	0.6	0.1 [0.19]	0.5	0.7	0.2 [<0.001]
Thyroid	2.3	2.1	-0.2 [<0.001]	2.8	2.6	-0.2 [<0.001]	2.8	2.4	-0.4 [<0.001]
Epilepsy	0.7	0.6	0.0 [0.91]	0.6	0.8	0.2 [<0.001]	0.5	0.9	0.4 [<0.001]

practice and population assigned data

Definitions for CHD indicators are given in Table 4. Table 5 shows the relationship between deprivation and population achievement for CHD quality indicators. Greater variation was found between England and Scotland for differences between the lowest and highest deprived deciles than was apparent with QOF prevalence. The difference between or the lowest and highest deprived deciles less than 0.2% points between England and Scotland for only three indicators (CHD03, CHD05, CHD08). The biggest differences were found for CHD10, where England was 0.3% points lower for the most deprived decile compared to 3.2% points lower in Scotland; CHD06, 1.5% points lower in England compared to no difference in Scotland; and CHD12, 4.2% points lower in England compared to 3.3% points lower in Scotland. Using practice population assigned data, differences increased for all the indicators with the exception of CHD10 where the difference between the least and most deprived deciles fell from 3.2% points to 2.0% points. The biggest difference using practice population assigned data was for CHD12 where the gap between the least and most deprived deciles increased from 3.3% points lower in the most deprived decile to 6.9% points lower. When practice postcode assigned data were used, there were significant differences between the least and most deprived deciles for two of the eight indicators, compared to significant differences for four indicators when practice population assigned data were used.

**Table 4:** Description of CHD QOF indicators used

**Disease area**	**Indicator definition**
CHD 03	Record of smoking status in the previous 15 months
CHD 05	Record of blood pressure in previous 15 months
CHD 06	Blood pressure recorded in previous 15 months ≤ 150/90
CHD 07	Record of total cholesterol in previous 15 months
CHD 08	Total cholesterol recorded in previous 15 months ≤ 5 mmol/l
CHD 09	Aspirin, alternative anti-platelet or anti-coagulant being taken
CHD10	Treated with beta-blocker
CHD12	Record of Influenza immunisation in previous flu season

**Table 5:** Differences between least and most deprived income deciles in CHD population achievement

	**England: Population achievement based on ** **practice postcode**			**Scotland: Population achievement based on ** **practice postcode**			**Scotland: Population achievement based on ** **practice population**		
	**Least** **deprived** ** decile**	**Most** **deprived** ** decile**	**Difference**	**Least** **deprived** ** decile**	**Most** **deprived** ** decile**	**Difference**	**Least** **deprived** ** decile**	**Most** **deprived** ** decile**	**Difference**
CHD03	95.8	96.0	0.2 [0.43]	97.1	97.1	0.0 [0.95]	97.4	97	-0.4 [0.33]
CHD05	97.6	97.1	-0.5 [<0.001]	97.2	96.6	-0.6 [0.15]	97.8	96.2	-1.6 [<0.001]
CHD06	85.1	83.6	-1.5 [<0.001]	86.6	86.6	0.0 [0.72]	86.7	86.4	-0.3 [0.88]
CHD07	91.5	90.9	-0.6 [0.03]	91.7	90.4	-1.3 [0.15]	92.3	90.3	-2.0 [<0.001]
CHD08	72.4	70.9	-1.5 [<0.001]	73.8	72.2	-1.6 [0.29]	74.7	71.7	-3.0 [<0.001]
CHD09	91.8	91.9	0.1 [0.72]	93.5	94.1	0.6 [0.30]	93.7	94.4	0.7 [0.11]
CHD10	51.7	51.4	-0.3 [0.57]	57.4	54.2	-3.2 [<0.001]	56.1	54.1	-2.0 [0.02]
CHD12	83.8	79.6	-4.2 [<0.001]	83.4	80.1	-3.3 [<0.001]	84.8	77.9	-6.9 [<0.001]

for practice and population assigned data

Table 6 shows the association between LLTI and population achievement for CHD quality indicators. For practice postcode assigned data, similar differences were found for England and Scotland across most of the indicators. The biggest difference between England and Scotland was for CHD12 where England was 3.6% points lower for the most deprived decile compared to 2.3% points lower in Scotland. Using practice population assigned data, differences between the deciles increased for all indicators compared to differences obtained using practice postcode assigned data. The biggest difference was found for CHD12, where the difference of 2.3% points lower using practice postcode assigned data increased to 7.5% points lower when practice population assigned data were used. For practice postcode assigned data, there were significant differences between the lowest and highest LLTI deciles for just one of the eight indicators, compared to four when practice population assigned data were used.

**Table 6:** Differences between lowest and highest LLTI deciles in CHD population achievement for

	**England: Population achievement scores ** **based on practice postcode**			**Scotland: Population achievement based on** **practice postcode**			**Scotland: Population achievement based on ** **practice population**		
	**Lowest** **LLTI** ** decile**	**Highest** **LLTI** ** decile**	**Difference**	**Lowest** **LLTI** ** decile**	**Highest** **LLTI** ** decile**	**Difference**	**Lowest** **LLTI** ** decile**	**Highest** **LLTI** ** decile**	**Difference**
CHD03	95.7	95.9	0.2 [0.47]	97.4	97.7	0.3 [0.39]	97.4	97.1	-0.4 [0.33]
CHD05	97.6	97.0	-0.6 [<0.001]	97.2	96.8	-0.4 [0.33]	97.7	96.3	-1.4 [<0.001]
CHD06	84.9	83.8	-1.1 [<0.001]	87.1	87.1	0.0 [0.94]	86.1	86.7	0.6 [0.46]
CHD07	91.4	90.9	-0.5 [0.04]	92.1	91.6	-0.5 [0.58]	92.6	90.6	-2.0 [<0.001]
CHD08	72.3	71.0	-1.3 [<0.001]	75.1	73.5	-1.6 [0.20]	75.3	72.4	-2.9 [<0.001]
CHD09	91.8	91.9	0.1 [0.75]	93.9	94.0	0.1 [0.81]	93.8	94.6	0.8 [0.08]
CHD10	51.6	51.2	-0.4 [0.34]	56.5	55.0	-1.5 [0.13]	57.2	54.1	-3.1 [0.02]
CHD12	83.2	79.8	-3.6 [<0.001]	82.8	80.5	-2.3 [0.01]	85.1	77.6	-7.5 [<0.001]

practice and population assigned data

## Discussion

This study has examined how the population level at which data are linked impacts on associations between QOF prevalence rates, achievement for CHD quality indicators and deprivation and ill health. We have found, through comparing Scottish data on IMD income domain and LLTI assigned at either practice postcode or practice population level, that analyses based on data assigned at practice postcode level under-estimated the relationship between deprivation and ill health for both prevalence and quality of care compared to what has been described as the 'gold standard' method of assigning deprivation and LLTI scores to practices based on the postcodes that the practice populations served live in[16]. Indeed, assigning income deprivation and LLTI scores at the level of the practice postcode exaggerated the variation across the deciles. The results of this study are consistent with the findings of a previous study based in one PCT in England, which found that using data assigned to the practice postcode underestimated the association between deprivation and ill health [16]. While the reason for this is unknown, it could be hypothesised that a contributory factor may be the positioning of the GP surgery itself, in relation to the population it serves, with practices located in areas significantly different to the areas that registered patients live. In addition, practice populations are often spread over a wide catchment area, which may not reflect the location of the surgery [22]. This may be exacerbated if the surgery has branch premises, as income deprivation and ill health were assigned to practise on the basis of the postcode of the main surgery.

For all but one of the indicators in this study, whether for prevalence or based on QOF achievement, the relationship between ill health and deprivation using data assigned to the practice postcode was under reported whether the relationship was a positive or negative one, although for the majority of indicators the difference between results was small. Differences between practice postcode and practice population depend on the level of variation explained by the dependant variable (income deprivation or LLTI) on the independent variable. The larger the amount of variation explained under practice population the bigger the difference. Generally, differences were larger for prevalence rates. So for COPD, practice postcode data based on income underestimated the difference between the least and most deprived deciles by 1.1% points, which was more than double the mean prevalence rate for COPD in Scotland of 1.9% points. As data are not available at practice population level nationally in England, we can only assume that given evidence from a previous study that it would show similar trends to Scotland [16].

This paper used deprivation measured at an aggregate level and therefore it is possible that associations identified here could differ if those associations were measured at the level of individuals, a concept referred to as the ecological fallacy or bias [23]. The use of data based on the practice population may help to alleviate some of the difficulties encountered from the use of aggregated data. Moreover, deprivation and health have been shown to have both area level and individual level factors [24] and, as such, the use of aggregated level data may be seen as an appropriate method [25]. The use of only one domain of the IMD, income, may also be seen as a limitation. However, as the correlation between income scores and total domain scores were almost perfectly correlated for both England and Scotland (R = 0.95 and 0.90 respectively), we anticipate that little or no difference would be found by using the total IMD score compared to income only. The income score also had the advantage of allowing a clear interpretation (the proportion of residents in receipt of state benefit on the grounds of low income).

A further limitation of this study concerns the calculation of prevalence rates. The Quality Management and Analysis System (QMAS), which releases QOF data in England, reports only raw prevalence figures for each condition, not age-sex standardised rates. Furthermore, as it is possible that as case identification rates may vary with deprivation this may lead to QOF prevalence rates underestimating levels in more deprived areas than in more affluent areas. The contribution of this potential confounder was explored using age-sex standardised prevalence rates from practices contributing to the Practice Team Information dataset collected by ISD Scotland [26] (see additional file 1 for methodology and results) which showed similar results to raw prevalence rates. We have also examined differences based on quality of care as measured by the QOF using what we have previously termed as population achievement [4]. While we recognise that there is debate over whether QOF indicators truly measure clinical quality, the indicators examined are all strongly evidence based and reflect current national clinical guidelines. The term population achievement has been used before both by the authors and other colleagues and allows for consistency in the description of the measurement of QOF indicators [4,27].

## Conclusion

In the absence of more detailed information, data based on the practice postcode is commonly used. In terms of the QOF there is a growing body of research examining how deprivation is associated with quality of care [5,7,8,13]. Evidence from this study suggests that this research may underestimate associations between deprivation and ill health, which should increase one's caution in interpreting such findings. Researchers in Scotland should make use of the data assigned to practice populations published by ISD Scotland. Strong and colleagues have suggested an alternative method of measuring deprivation, using Geographical Information Systems (GIS)[15]. This approach was demonstrated to have a better agreement with deprivation based on the practice population compared to the practice postcode method. Therefore it may be of use to researchers both in England and elsewhere who do not have access to patient population data. However, the GIS method still overestimates deprivation in more deprived areas and underestimates in less deprived areas compared to the practice population method [15]. Given the importance of understanding the effect of deprivation and ill health on a range of determinants related to health care policy makers should seek to ensure that practice population data is available at national level in England.

## Methods and Data

We used publicly available data on QOF achievement and prevalence for each practice in England and Scotland for the period 2005–06 [17,18]. The unadjusted prevalence rates were calculated for each individual domain by dividing the number of patients on the disease register by the practice population and multiplying by 100.

For our measure of QOF achievement we used population achievement (based on the care delivered to all patients) for CHD quality indicators where the denominator was all patients with the disease. This method has been explained in more detail elsewhere and does not remove those patients 'exception reported' from the numerator population [6]. CHD was chosen as it is a national priority in both countries and is the QOF domain with the highest number of points attached to it (121) representing 22% of the total points available for clinical indicators (Table 4).

Deprivation for England and Scotland was measured using the income domain of the Index of Multiple Deprivation (IMD) from 2004 for each country [19,20]. The income domain was used rather than the overall score or other domains, because it is the only domain calculated in a similar way in both countries. In both countries, the income domain contributes, jointly with employment, the highest proportion of the overall index (22.5% in England, 29.0% in Scotland) and is highly correlated with the overall IMD score (R = 0.95 and 0.99 for England and Scotland respectively). The income domain reports on the percentage of patients receiving state benefits on the basis of low income. Thus, the higher the reported score the more income deprived the practice population is. For practice postcode assigned deprivation, deprivation was based on the income score of the practice postcode calculated by linking the postcode of the practice's main surgery to its Census Lower Layer Super Output Area (LSOA) for England and datazone level for Scotland, and then to its IMD domain score [19]. For Scotland deprivation was also assigned at practice population level, based on the mean score of the registered practice population. Data on the practice population scores were obtained from ISD Scotland [21].

Health for England and Scotland was measured using data from the 2001 Census on the level of limiting long-term illness (LLTI) for both countries. An age-sex standardised ratio was calculated for LLTI and a practice score assigned using the same procedure as for deprivation. For Scotland, data were obtained from ISD Scotland at both the level of the practice postcode and based on the mean score of the registered practice population. Prevalence data indirectly age and sex standardised using data from PTI practices obtained from ISD Scotland [see Additional file 1]. Expected prevalence figures for each practice were calculated by applying PTI-based age-sex specific rates to practice population counts by age and gender. Standardised prevalence rates were then obtained by dividing the actual prevalence figures reported in QMAS by these expected figures. The resultant standardised rates were centred on a Scottish average of 100.

Data were available in total for 8167 English and 989 Scottish practices (97% and 98% of the total number of practices respectively). Practices were divided into deciles based on income and levels of limiting long-term illness and weighted by population size. We compared the mean prevalence rates for the ten QOF clinical domains and the achievement scores for the eight CHD QOF indicators for (a) practices in the least and most deprived deciles, as measured by the income domain and (b) practices with the lowest and highest LLTI deciles. Significance testing was based on practice-level data using a threshold of p < 0.01 as our measurement of significance. The analysis was undertaken in STATA v8.2, using robust standard errors. The calculation of mean values and the regression coefficients were weighted by population size.

## Competing interests

The authors declare that they have no competing interests.

## Authors' contributions

GM had the original idea, and planned the analysis with all the other authors. GM conducted the data analysis and all authors wrote and revised the manuscript. GM is the guarantor.

## Supplementary Material

Additional file 1 Age-sex standardised results for QOF prevalence rates between the least and most deprived deciles for practice and population assigned data. The data represent the age and sex standardised results for QOF prevalence rates for the least and most deprived deciles. Click here for file
