# Supplementary material for: Practice postcode versus patient population: a comparison of data sources in England and Scotland
Source: Int J Health Geogr. 2008 Jul 16;7:37. doi: 10.1186/1476-072X-7-37 (PMC2490685; doi:10.1186/1476-072X-7-37)
Supplement: Additional file 1 — Age-sex standardised results for QOF prevalence rates between the least and most deprived deciles for practice and population assigned data. The data represent the age and sex standardised results for QOF prevalence rates for the least and most deprived deciles. [file 1476-072X-7-37-S1.doc]

# Additional Table 1: Age-sex standardised results for QOF prevalence rates between the least and most deprived deciles for practice and population assigned data

|  | **Scotland: Prevalence rates based on practice**  **postcode** | | | **Scotland: Prevalence rates based on practice**  **population** | | |
| --- | --- | --- | --- | --- | --- | --- |
|  | **Least**  **deprived**  **decile** | **Most**  **deprived**  **decile** | **Difference** | **Least**  **deprived**  **decile** | **Most**  **deprived**  **decile** | **Difference** |
| CHD | 89.67 | 115.69 | 26.03 [<0.001] | 77.38 | 126.01 | 48.63 [<0.001] |
| Diabetes | 91.12 | 112.71 | 21.59 [<0.001] | 78.00 | 119.55 | 41.56 [<0.001] |
| Stroke | 93.56 | 114.44 | 20.88 [0.03] | 84.00 | 125.04 | 41.04[<0.001] |
| BP | 96.09 | 103.96 | 7.87 [0.27] | 90.50 | 110.21 | 19.71 [0.06] |
| COPD | 76.65 | 153.36 | 76.71 [<0.001] | 49.91 | 194.37 | 144.46 [<0.001] |
| Asthma | 96.68 | 100.98 | 4.30 [0.68] | 91.74 | 104.06 | 12.32 [0.11] |
| Thyroid | 96.35 | 91.77 | -4.58 [0.34] | 101.31 | 86.89 | -14.43 [0.09] |
| Epilepsy | 89.43 | 121.02 | 31.59 [<0.001] | 75.64 | 133.39 | 57.75 [<0.001] |
